# Threatening Life Events and Difficulties and Psychotic Disorder

**DOI:** 10.1093/schbul/sbaa005

**Published:** 2020-02-12

**Authors:** Stephanie Beards, Helen L Fisher, Charlotte Gayer-Anderson, Kathryn Hubbard, Ulrich Reininghaus, Thomas J Craig, Marta Di Forti, Valeria Mondelli, Carmine Pariante, Paola Dazzan, Robin Murray, Craig Morgan

**Affiliations:** 1 Social Epidemiology Research Group, Health Service and Population Research Department, Institute of Psychiatry, Psychology & Neuroscience, King’s College London, London, UK; 2 MRC Social, Genetic & Developmental Psychiatry Centre, Institute of Psychiatry, Psychology & Neuroscience, King’s College London, London, UK; 3 ESRC Centre for Society and Mental Health, King’s College London, London, UK; 4 Central Institute of Mental Health, Medical Faculty Mannheim, University of Heidelberg, Mannheim, Germany; 5 Psychosis Studies Department, Institute of Psychiatry, Psychology & Neuroscience, King’s College London, London, UK

**Keywords:** life events, difficulties, intrusiveness, psychotic disorder

## Abstract

**Objective:**

Stressful life events have been implicated in the onset of psychotic disorders, but there are few robust studies. We sought to examine the nature and magnitude of associations between adult life events and difficulties and first-episode psychoses, particularly focusing on contextual characteristics, including threat, intrusiveness, and independence.

**Method:**

This study forms part of the Childhood Adversity and Psychosis Study (CAPsy), an epidemiological case-control study in London, United Kingdom. Data on life events and difficulties (problems lasting 4 wk or more) during 1 year prior to onset (cases) or interview (controls) were assessed using the semi-structured Life Events and Difficulties Schedule (LEDS). Data were available on 253 individuals with a first episode of psychosis and 301 population-based controls.

**Results:**

We found strong evidence that odds of exposure to threatening and intrusive events in the 1 year prior to onset were substantially higher among cases compared with controls, independent of age, gender, ethnicity, and social class (ORs > 3). This was consistent across diagnostic categories. We found further evidence that the effect of threatening events and difficulties was cumulative (1 event odds ratio [OR] 2.69 [95% confidence interval (CI) 1.51–4.79]; 2 events OR 4.87 [95% CI 2.34–10.16]; ≥3 events OR 5.27 [95% CI 1.83–15.19]; 1 difficulty OR 3.02 [95% CI 1.79–5.09]; 2 difficulties OR 9.71 [95% CI 4.20–22.40]; ≥3 difficulties OR 12.84 [95% CI 3.18–51.85]).

**Conclusions:**

Threatening and intrusive life events and difficulties are common in the year pre-onset among individuals with a first episode of psychosis. Such experiences may contribute to the development of psychotic disorders.

## Introduction

Life events have long been postulated as precipitants of psychoses.^1^ In our meta-analysis,^2^ we found that exposure to recent life events was around 3 times more common among cases with psychosis than controls (weighted summary odds ratio [OR] 3.19; 95% confidence intervals [CI] 2.15–4.75). However, the number of studies was small (*n* = 11) and many had notable methodological limitations (eg, small sample sizes, mixed first-episode and non–first-episode samples, absent or poorly selected control group, and checklist measures of life events), with no consideration of contextual influences on the meaning and interpretation of events.

There are other methodological issues that limit what can be inferred from previous research. For example, it is possible that the higher reported prevalence of life events in patients with psychosis is an artifact of unreliable reporting, possibly influenced by the individual’s attempt to find an explanation for their disorder.^3,4^ However, few studies to date have taken any steps to address the potential influence of recall bias (eg, by carefully eliciting detailed accounts, using life course methods such as anchoring by key dates, to aid recall). Another possibility is that more events occur in the period prior to full onset due to the insidious development of symptoms or premorbid characteristics. One way to address this is to distinguish between events that are independent of emerging symptoms (eg, death of a close relative) and those that may be influenced by a deteriorating mental state (eg, interpersonal conflict). Some studies have found that even when only independent events are considered there remains evidence of an association with psychosis.^1,5–7^

Further, few studies have considered other aspects of life events and difficulties (ie, problems occurring over time), including their type and severity. It is possible, for example, that more severe^6,7^ and intrusive^8^ life events are particularly important in the development of psychoses, possibly via effects on the development of negative cognitive schema about others and on biological systems and processes implicated in psychoses (eg, HPA axis, dopamine system, inflammation). Brown and Harris usefully defined severe and threatening events as those likely to produce a strong emotional reaction, and intrusive events are those that involve unwanted interference and/or attempted control of an individual’s personal boundaries, usually by people or organizations outside of the individual’s personal network (eg, burglaries, physical attacks, visits by the police).^8^ In a study of 50 cases with psychosis and 238 population-based controls, Harris^8^ found that individuals with schizophrenia were nearly 20 times more likely than controls to report exposure to intrusive events in the 3 weeks pre-onset or relapse (10 [20%] cases exposed to intrusive events vs 3 [1%] controls; OR 19.58; 95% CI 5.16–74.27). Interestingly, there is also evidence that exposure to intrusive events in childhood involving clear intent to harm are associated with increased risk of later low-level psychotic or anomalous experiences.^9,10^

Using detailed data on life events from the Childhood Adversity and Psychosis study, we sought to test the hypotheses that exposure to threatening and intrusive life events and difficulties, in the period immediately before onset, are associated with increased odds of all psychoses and that these associations also hold for independent events and difficulties.

## Method

### Design

The Childhood Adversity and Psychosis (CAPsy) study is a population-based case-control study of first-episode psychosis, which was conducted over a 4-year period (Jan 2010–Jan 2014). The study was designed to investigate the relationship between adversities across the life course and psychotic disorder, focusing on the timing, duration, and severity of exposures, on interactions with other risk factors (eg, substance use), and putative psychological and biological mechanisms.

### Sample (1): Cases

Our inclusion criteria for cases were: age 18–64 years, resident within a defined catchment area in south-east London, United Kingdom, presence of a first-episode psychotic disorder (ie, International Classification of Disease [ICD] diagnoses F20-29 and F30-33) within the time frame of the study, and no previous contact with mental health services for psychosis. Exclusion criteria were: evidence of psychotic symptoms with an organic cause, transient psychotic symptoms resulting from acute intoxication as defined by ICD-10; severe learning disabilities; and insufficient understanding of English to complete the assessments.

To identify potential cases, a team of researchers regularly (ie, at least weekly) screened both general adult and specialist inpatient, outpatient, and community services in the catchment areas. All potential cases were screened for inclusion using the Screening Schedule for Psychosis.^11^ When considered appropriate by mental health staff, all those who met the inclusion criteria were approached and informed consent sought. In total, 599 potential cases were identified during the study period. Of these, 374 (62.4%) consented and were assessed. We were not able to collect any information on those who could not be contacted or who refused. However, we were able to compare the basic characteristics of cases who consented with those from a concurrent case-register–based incidence study of all individuals with a first-episode psychosis in our catchment areas (see supplementary table 1). On basic characteristics, our sample included more men and was, on average, younger.

### Sample (2): Controls

A population-based and demographically representative sample of controls resident in our catchment areas, aged 18–64 years, and without a current or past history of psychotic disorder was recruited using a mixture of quota and random sampling. First, quotas were set for gender, age group, and ethnic group. The quotas for each group were set to ensure a sample of controls that reflected the demographic profile, based on the 2011 Census, of the local population and that included a sufficient number of controls from black Caribbean and black African groups for potential analyses by ethnic group. Second, 2 sampling frames were used to fill these quotas: (*a*) the UK postal address file (PAF) and (*b*) general practitioner (GP) lists. First, the Royal Mail Small Users PAF^12^ provides a list of all households in the United Kingdom. We used this to randomly select addresses within the catchment area. The selected addresses were sent letters of invitation to take part and, then, at least 2 weeks later, each address was visited on at least 4 separate occasions at different times of the day (morning, afternoon, and evening) and on different days of the week (including weekends). Residents were given written and verbal information concerning the study and were asked whether anyone in the household might be eligible and interested in taking part. If all potential controls within the household refused, or no members were eligible, then the next address on the PAF list was visited. A total of 695 letters were sent; 326 potential controls were identified; and 133 (44.2%) were selected (ie, fit one of our quotas), recruited, and assessed. Second, in the catchment areas, 12 GP surgeries were randomly selected and, from the lists of each of these, 3600 individuals who met the inclusion criteria for controls were randomly selected and sent letters of invitation to take part. A total of 515 responded to the invitation and 168 (55.8%) were selected (ie, fit one of our quotas), recruited, and assessed. All potential controls were screened for a current or past history of psychosis using the Psychosis Screening Questionnaire.^13^

### Data Collection

All cases and controls completed a series of interviews and assessments that elicited information on a wide range of clinical (eg, symptoms, diagnosis, duration of untreated psychosis, premorbid adjustment), social (eg, sociodemographic characteristics, childhood adversities, adult life events, social support and networks), neuro- and social cognition, and biological (eg, cortisol, MRI, DNA) variables.

### Life Events and Difficulties

Information on life events and difficulties in the year pre-onset or, for controls, pre-interview was obtained using the semi-structured Life Events and Difficulties Schedule (LEDS).^14^ The LEDS has good psychometric properties, with established reliability (eg, levels of inter-rater agreement of 80% or more) and validity (eg, high levels of agreement between different informants),^15,16^ and has been successfully used with individuals with psychosis.^1,5–7^ The LEDS distinguishes between discrete events and ongoing difficulties (ie, problematic situations that last for 4 wk or more). All life events and difficulties were rated for their level of threat, intrusiveness, and independence. *Threat* is a general indicator of the likely stressfulness of the experience. This and other ratings of the meaning of experiences are investigator-based and contextual (ie, taking account of how an average person with similar biography and circumstances would be expected to feel in response to the stressor, ignoring any reported emotional response). For events, threat is rated on a 4-point scale (marked, moderate, some, little or none) and, for difficulties, on a 7-point scale (high marked, low marked, high moderate, low moderate, mild, very mild, not/no longer a difficulty). For analyses, these scales were dichotomized into marked/moderate vs mild/some/none, in line with previous studies.^5,17^ The contextual *intrusiveness* of events and difficulties was similarly rated on a 4-point scale (ie, marked intrusiveness, moderate intrusiveness, some intrusiveness, little or no intrusiveness) and dichotomized as above. All events and difficulties were rated as either independent (ie, unlikely to be influenced by any [developing] psychotic disorder) or possibly dependent (ie, could have been influenced by any [developing] psychotic disorder). Using detailed notes taken during interviews, all LEDS ratings were subsequently made by consensus within the research team, a painstaking process designed to increase accuracy and consistency of ratings for all interviews. Examples of ratings are provided in Appendix 1 (see Supplementary Material).

### Demographic and Clinical Data and Putative Confounders

An amended Medical Research Council (MRC) Sociodemographic Schedule^18^ was used to collect data on age at interview, gender, ethnicity, and participants’ main social class (classified into 6 classes using the European Socio-Economic Classification system).

The Operational Criteria Checklist for Psychotic and Affective Disorders (OPCRIT)^19^ was used to derive Diagnostic and Statistical Manual (DSM)-IV/ICD-10 diagnoses for cases. The checklist was completed based on data collected with the SCAN^20^ and case records for the month following the first contact with mental health services for psychotic symptoms. Diagnoses were dichotomized into 2 main categorical groups: (1) non-affective psychosis (ICD-10 codes F20-29, including diagnoses of schizophrenia, schizoaffective disorder, delusional disorders, and psychosis Not otherwise specified) and (2) affective psychosis (ICD-10 codes F30-F33, including diagnoses of bipolar disorder, mania, and depressive disorders). The Nottingham Onset Schedule (NOS)^21^ was used to estimate the date of onset of psychosis, defined as the time when there was clear evidence of positive psychotic symptoms (ie, a score of at least 2, indicating clinically meaningful, for a psychotic item in Part II of the SCAN).^20^

We further collected data on 3 putative confounders: proxy genetic risk, premorbid adjustment, and cannabis use. The Family Interview for Genetic Studies (FIGS)^22^ was used to collect information about the participant’s family history of mental illness and was included as an indirect measure of genetic risk. The Premorbid Adjustment Scale (PAS)^23^ was used to measure premorbid personality on a 7-point scale, with overall mean scores (0 healthiest adjustment to 6 lowest adjustment) calculated for academic adjustment and social adjustment. The Cannabis Experience Questionnaire (Modified version^24^) was used to collect information on current and past cannabis use. For the present analyses, current use was defined as at least a single use of cannabis in the last year.

### Analyses

We used binary and multinomial logistic regression to quantify, using unadjusted and adjusted ORs, associations between life events and difficulties and psychosis (overall and by diagnosis), taking account of severity, intrusiveness, and independence. Multinomial logistic regression was used to examine associations by diagnostic category. All analyses were adjusted, first, for gender, age, ethnicity, and social class (ie, partially adjusted model), and, second, for family history, premorbid adjustment, and cannabis use (ie, fully adjusted model). In handling missing data, all analyses were complete data analyses. In addition, all analyses were weighted to take account of oversampling of black Caribbean and black African controls. Analyses were conducted in STATA (version 14).^25^

### Ethics

Ethical approval for this study was obtained from the South London and Maudsley (SLaM) NHS Foundation Trust and the Institute of Psychiatry, Psychology & Neuroscience (IoPPN) Research Ethics Committee Ref: 321/05, including amendments 1–9. After a complete description of the study was given to the participants, written informed consent was obtained.

## Results

We identified, recruited, and assessed 374 cases and 301 controls. Of these, 253 (68%) cases and 301 (100%) controls completed a LEDS interview. Reasons for incompletion were: completion of the assessment battery before the LEDS was added (*n* = 42); drop out (*n* =60), childhood onset (ie, prior to 17 years; *n*= 16), refusal (*n* = 1), and mental state (*n* = 2). When we compared the 121 cases who did not complete a LEDS interview with the 253 cases who did, there were no substantial differences by age, gender, and diagnosis (supplementary table 2).

### Sample Characteristics

In line with what we would expect, compared with controls, cases were younger and more often men, of nonwhite ethnicity, and poorly educated; less likely to be in the highest social class group; and more likely to currently use cannabis and have a first-degree relative with psychosis (table 1). The majority of cases (complete data for 250 cases) had a diagnosis of non-affective psychotic disorder (*n* = 190, 76.0%). Broadly, our sample included more men and was, on average, younger than the concurrent incidence study sample (supplementary table 1).

**Table 1.** Sociodemographic Characteristics of the Life Events Sample

	Cases (*n* = 253)	Controls (*n* = 301)			
	Mean (SD)	Weighted Mean (SD)	*T*	*df*	*P*
Age in years	29.0 (8.85)	37.0 (12.20)	−8.44	553	**<.001**
	*n* (%)	*n* (w%)	*x* ^2^	*df*	*P*
Sex					
Men	156 (61.7)	153 (50.1)	7.41	1	**.011**
Women	97 (38.3)	148 (49.9)			
Ethnicity					
White British	70 (27.7)	131 (42.6)	39.15	5	**<.001**
White Other	32 (12.7)	44 (21.8)			
Black African	65 (25.7)	50 (13.0)			
Black Caribbean	45 (17.8)	44 (11.2)			
Asian (all)	13 (5.1)	17 (6.8)			
Other	28 (11.1)	15 (4.7)			
Highest level of education (6 missing values)					
University	56 (22.5)	165 (56.7)	75.81	2	**<.001**
Further education	104 (41.8)	96 (31.1)			
School	89 (35.7)	38 (12.3)			
Subject social class (main)					
Salariat	28 (11.1)	150 (53.8)	150.18	5	**<.001**
Intermediate	71 (28.1)	76 (25.6)			
Working Class	103 (40.7)	37 (10.7)			
Student	20 (7.9)	32 (7.8)			
Long-term unemployed	22 (8.7)	1 (0.2)			
Non-classifiable	9 (3.6)	5 (1.9)			
Family history of psychosis (78 missing values)					
No	182 (85.9)	252 (93.6)	7.85	1	**.044**
Yes	30 (14.2)	12 (6.4)			
Current cannabis use (50 missing values)					
No	155 (67.7)	230 (83.6)	17.48	1	**<.001**
Yes	74 (32.3)	45 (16.4)			
Diagnosis—cases only (3 missing values)					
Non-affective psychosis	190 (76.0)	—	—	—	—
Affective psychosis	60 (24.0)	—			

*Note*: *df*, degrees of freedom; SD, standard deviation. w, weighted (for the population proportions of age, gender and ethnicity according to Census values within Lambeth & Southwark).

### Life Events and Difficulties

**Fig. 1.**
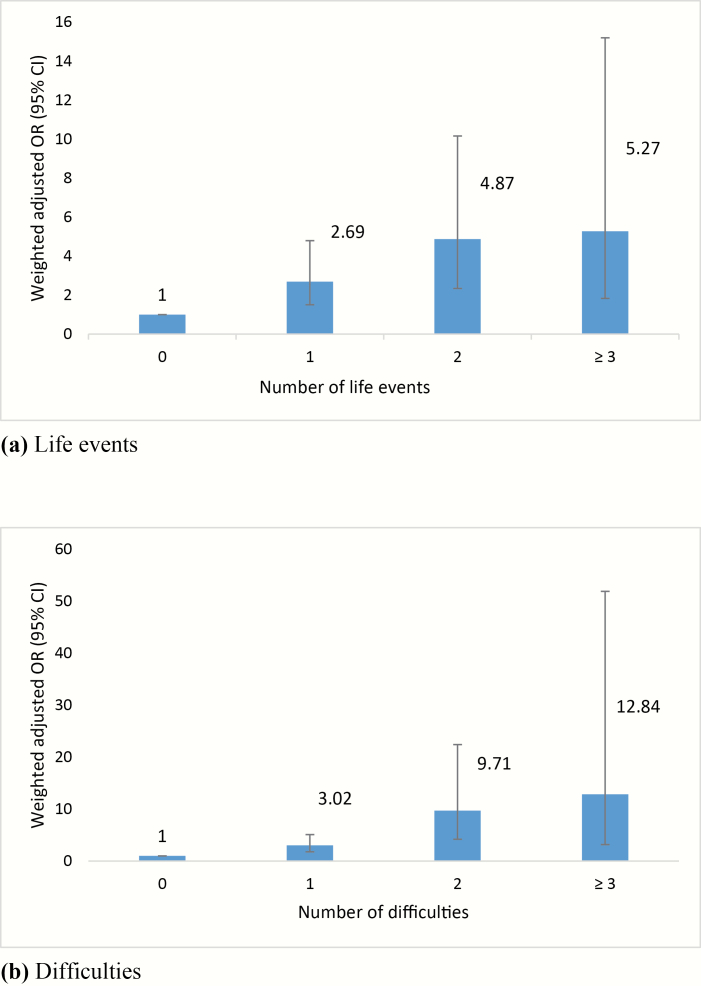
Cumulative associations between case-control status and moderate/marked (a) life events and (b) difficulties. Weighted odds ratio (OR) with 95% confidence intervals (CI), adjusted for age, gender, ethnicity, social class. Analyses were weighted for the population proportions of age, gender, and ethnicity according to Census values within Lambeth & Southwark.

Cases were around 3-times more likely than controls to report exposure to at least one moderate or marked threatening event in the year prior to onset (cases 48.6% vs controls 21.5%; partially adjusted OR 3.52, 95% confidence interval [CI] 2.20–5.64; table 2). Further, there was some evidence that the odds of psychosis increased with each additional life event (figure 1a; ie, 1 event: partially adjusted OR 2.69, 95% CI 1.51–4.79; 2 events: partially adjusted OR 4.87, 95% CI 2.34–10.16; 3 events: partially adjusted OR 5.27, 95% CI 1.83–15.19), albeit with wide CIs. When modeled as a count variable, the odds of psychosis increased by around 80% for each additional life event (partially adjusted OR 1.84, 95% CI 1.45–2.33).

**Table 2.** Association Between Moderate/Marked Life Events and Difficulties and Psychotic Disorder

	Cases (*n* = 253) *n* (%)	Controls (*n* = 301) *n* (w%)	Unadjusted OR (95% CI)	Adjusted OR^a^ (95% CI)
Event				
None	130 (51.4)	237 (78.5)	1	1
1 or more	123 (48.6)	64 (21.5)	3.45 (2.30–5.15)*	3.52 (2.20–5.64)*
Difficulty				
None	117 (46.3)	227 (74.4)	1	1
1 or more	136 (53.6)	74 (25.6)	3.38 (2.29–4.97)*	4.69 (2.93–7.53)*

*Note*: w, weighted (for the population proportions of age, gender, and ethnicity according to Census values within Lambeth & Southwark); OR, odds ratio, calculated using weighted data; CI, confidence interval.

^a^Adjusted for age, gender, ethnicity, social class (Percentages may not add up to 100 due to rounding).

*P < .001.

As with life events, cases were more likely than controls to report exposure to at least one moderate or marked threatening difficulty (cases 53.6% vs controls 25.6%) (table 2); that is, reported difficulties were associated with 4-fold increased odds of psychosis (partially adjusted OR 4.69, 95% CI 2.92–7.53). Again, as with events, there was some evidence that the odds of psychosis increased with each additional difficulty (see figure 1b; ie, 1 difficulty: partially adjusted OR 3.02, 95% CI 1.79–5.09; 2 difficulties: partially adjusted OR 9.71, 95% CI 4.20–22.40; 3 difficulties: partially adjusted OR 12.84, 95% CI 3.18–51.85), albeit with wide CIs. When modeled as a count variable, the odds of psychosis increased by around 2½ times for each additional difficulty experienced (partially adjusted OR 2.51; 95% CI 1.89–3.32).

There was no evidence that the magnitude of associations between threatening life events, difficulties, and psychosis differed between cases with a non-affective disorder and cases with an affective disorder (supplementary tables 3 and 4).

### Intrusiveness

More specifically, cases were more likely to report exposure to moderate or marked intrusive events in the year prior to onset compared with controls (cases 19.0% vs controls 2.8%). The effect of intrusive events was greater than the effect of nonintrusive events (table 3). That is, intrusive events were associated with a 6-fold increased odds of psychosis (partially adjusted OR 6.58, 95% CI 2.81–15.44), compared with around a 3-fold increased odds for nonintrusive events (partially adjusted OR 2.92, 95% CI 1.74–4.90). There were similar effects for intrusive difficulties (cases 10.3% vs controls 0.7%), with increased odds of psychosis of around 17, albeit with very wide CIs (partially adjusted OR 17.02, 95% CI 4.10–70.80). This was greater than for nonintrusive difficulties (partially adjusted OR 4.10, 95% CI 2.51–6.72).

**Table 3.** Association Between Moderate/Marked Life Event and Difficulty Intrusiveness and Psychotic Disorder

	Cases (*n* = 253) *n* (%)	Controls (*n* = 301) *n* (w%)	Unadjusted OR (95% CI)	Adjusted OR^a^ (95% CI)
Event				
None	130 (51.4)	237 (78.5)	1	1
Nonintrusive event(s)	75 (29.6)	54 (18.7)	2.42 (1.55–3.77)*	2.92 (1.74–4.90)*
Intrusive event(s)	48 (19.0)	10 (2.8)	10.22 (4.71–22.16)*	6.58 (2.81–15.44)*
Difficulty				
None	117 (46.3)	227 (74.4)	1	1
Nonintrusive difficulties	110 (43.5)	72 (24.9)	2.81 (1.89–4.19)*	4.10 (2.51–6.72)*
Intrusive difficulties	26 (10.3)	2 (0.7)	23.23 (5.30–101.86)*	17.02 (4.09–70.80)*

*Note*: Abbreviations are explained in the first footnote to Table 2.

^a^Adjusted for age, gender, ethnicity, social class (Percentages may not add up to 100 due to rounding).

*P < .001.

When we repeated analyses and further adjusted for a family history of psychosis, current cannabis use, and premorbid adjustment in the subsample on which we had complete data (*n* = 448; 178 cases and 270 controls), there was no evidence in these fully adjusted models that the associations could be explained by these other factors (see supplementary table 5).

### Independence

Overall, the effect of independent events (partially adjusted OR 2.99, 95% CI 0.99–9.07) was similar to that for nonindependent events (partially adjusted OR 3.62, 95% CI 2.21–5.92). Likewise, the magnitude of the effect of independent difficulties (ie, partially adjusted OR 3.98, 95% CI 0.63–25.09) was similar to that of nonindependent difficulties (ie, partially adjusted OR 4.73, 95% CI 2.95–7.59). However, numbers who reported independent events and difficulties, overall, were small and the estimated effects are consequently imprecise (table 4).

**Table 4.** Association Between the Independence of Moderate/Marked Life Events and Difficulties and Psychotic Disorder

	Cases (*n* = 253) *n* (%)	Controls (*n* = 301) *n* (w%)	Unadjusted OR (95% CI)	Adjusted OR^a^ (95% CI)
Event				
None	130 (51.4)	237 (78.5)	1	1
Nonindependent event(s)	111 (43.9)	50 (16.7)	4.01 (2.60–6.17)*	3.62 (2.21–5.92)*
Independent event(s) only	12 (4.7)	14 (4.8)	1.50 (0.63–3.59)	2.99 (0.99–9.07)
Difficulty				
None	117 (46.3)	227 (74.4)	1	1
Nonindependent difficulties	130 (51.4)	63 (22.2)	3.72 (2.48–5.58)*	4.73 (2.95–7.59)*
Independent difficulties only	6 (2.4)	11 (3.4)	1.12 (0.40–3.16)	3.98 (0.63–25.09)

*Note*: Abbreviations are explained in the first footnote to Table 2.

^a^Adjusted for age, gender, ethnicity, social class (percentages may not add up to 100 due to rounding).

*P < .001.

## Discussion

A number of notable findings emerged from these analyses. First, we found strong evidence that reported exposure to threatening life events and difficulties prior to onset was associated with around a 3- to 4-fold increased odds of psychosis (irrespective of broad diagnosis), with further evidence that the effect was cumulative. Second, the effect was strongest for events and difficulties characterized by intrusiveness, ie, those involving an element of control and/or intention to harm (eg, imprisonment, physical assault, rape). Finally, associations held when we considered only independent events and, more tentatively, difficulties. These analyses are, as far as we are aware, the first based on data from a sample of patients with first-episode psychosis compared with a population-based control group, and the largest case-control study to use the comprehensive LEDS interview,^14^ with careful dating of onset and adjustment for family history, premorbid personality, and cannabis use.

### Methodological Considerations

Before considering these findings further, several methodological issues need to be considered. Selection and recruitment of cases and controls could have biased findings and over-inflated differences if this led to preferential inclusion of cases with more events and/or controls with fewer events. To minimize bias in the selection and recruitment of cases, considerable effort was made to identify and recruit a representative case sample who presented to mental health services within our catchment area during the time frame of the study. However, not all those who were identified could be approached or consented. This could have biased the findings if, for example, those who were missed experienced fewer life events.

Conversely, selection bias may have occurred if controls who took part were less likely to experience life events. To minimize selection bias, we used a mixture of quota and random sampling to generate a representative sample, using 2 sampling frames. The combination of 2 sampling frames and methods was intended to minimize the biases associated with using each alone (eg, use of household survey methods tends to oversample those who are at home, unemployed, women with young children). The overall control sample was broadly representative of the population living within the catchment boroughs. Further, controls were not excluded if they had a psychiatric diagnosis other than psychosis, which ensured that the differences between cases and controls were not overinflated by including only well controls.^26^

Given information on life events and difficulties were collected retrospectively, recall bias may also have influenced our findings.^27^ To address the possibility of recall bias, all life events and difficulties were rated contextually to minimize the participant’s subjective view of threat and other characteristics of the reported experiences. Dates were also related to “anchoring points,” such as birthdays and national holidays, to increase accuracy.

Further, we cannot rule out the possibility that associations were a consequence of reverse effects (ie, of developing psychosis increasing exposure to life events and difficulties). We did, however, take steps to minimize this possibility. First, to establish temporal ordering, the date of onset was assessed before the LEDS interview was conducted. Accuracy was increased by using a combination of participant interviews and case notes and by using a measure with established reliability and validity, the NOS.^21^ Second, we sought to distinguish experiences which were unlikely to be the result of developing psychosis (independent events); in doing this, there was still some evidence of an association with psychosis, albeit the small number of participants who reported such events means that our estimated effects were imprecise and, therefore, need to be considered cautiously. All of this noted, attempting to draw this distinction may be misleading. Events may not simply be either a cause or consequence of (emerging) psychosis; rather, each may compound the other, creating a vicious cycle that, over time, pushes some along a pathway to psychotic disorder. However, at present, this is speculative and the possibility that associations between life events and psychosis reflect reverse effects remains.

### Life Events and Difficulties and Psychoses

The above limitations notwithstanding, our findings provide evidence of an association between threatening and intrusive experiences and the onset of psychosis. In line with this, previous first-episode studies have found similarly large associations (ORs of 3.2–5.0), particularly for events above a certain threat level.^1,5–7^ This includes 2 other first-episode studies that used the LEDS.^6,7^ Many studies have found that the percentage of cases exposed to threatening events prior to onset is roughly 50%,^1,5,28,29^ which closely resembles what we found in this study (48.6%). The evidence for a cumulative effect of threatening life events and difficulties is also in line with what has been found for childhood adversity and psychosis.^30–32^ Our work adds to and extends this by suggesting that associations extend to difficulties (not just discrete events) and are independent of putative confounders, including a family history of psychosis, premorbid adjustment, and cannabis use. It is also notable that associations between severe experiences and psychosis were evident across diagnostic groups.^5^

Very few studies have considered whether specific types of events are associated with psychosis. We found that intrusive events and difficulties were especially common prior to a first episode of psychotic disorder; these findings mirror some previous studies.^7,8,33^ Further, it is notable that the quality of intrusiveness (ie, unwanted interference and/or attempted control by others) also characterizes other experiences that have been linked to psychosis, eg, sexual abuse^34^ and severe bullying.^35^ In line with this, Arseneault et al,^9^ in a prospective study of around 2000 twins followed to age 12, found that experiences specifically involving an intention to harm (ie, bullying, maltreatment) but not others (ie, accidents) were associated with an increased risk of low-level psychotic experiences.

It may be that all difficult experiences impact to some extent, via shared mechanisms, on most mental health problems. These findings tentatively suggest that certain types of experience may also specifically increase odds of particular problems and disorders (ie, those involving threat and violence increases the likelihood of paranoia, hallucinations), as has been shown for humiliation and loss in depression.^36^

### Mechanisms

There are plausible psychological and biological mechanisms through which life events and difficulties may contribute to the development of psychoses.^37^ For example, early and later exposure to adverse life events may combine to increase the risk of psychosis^38,39^ via cognitive and affective processes highlighted by cognitive models of psychosis.^40,41^ As an illustration, negative schematic beliefs formed early in life as a consequence of maltreatment^42–44^ may be (re)activated if an individual experiences adversity in adulthood. These schemas may influence how individuals appraise their social worlds and create a tendency to perceive the external world as hostile, which over time may push some individuals along a continuum from suspiciousness to paranoia and ultimately persecutory delusions. Alongside, or further underpinning, this especially threatening events and difficulties may activate or exacerbate interconnected biological processes (eg, HPA axis activation, inflammation, sensitization of the dopamine system) implicated in the emergence of psychotic disorders.^45–47^

### Further Research

Our study provides robust data on associations between life events and difficulties and psychoses. However, there remain intrinsic limitations to the causal inferences that can be drawn from case-control data, and further research is required to both replicate our findings and extend them using designs that may allow stronger causal inferences (eg, existing cohort and population register data, quasi-experimental approaches using innovative methods, such as experience sampling). Further research is also needed that tests putative mechanisms, both to strengthen our understanding of possible pathways linking adverse experiences and psychoses and to enable more specific and targeted interventions to mitigate the effects of threatening events and difficulties among those most at risk.

## Funding

This work was supported by the Wellcome Trust (grant No. WT087417), the European Union (European Community’s Seventh Framework Program (grant agreement No. HEALTH-F2-2009–241909): Project EU-GEI), the UK Department of Health via the National Institute for Health Research (NIHR) Specialist Biomedical Research Centre for Mental Health award to the South London and Maudsley NHS Foundation Trust (SLaM) and the Institute of Psychiatry Psychology & Neuroscience, King’s College London, and the ESRC Centre for Society and Mental Health at King’s College London (ESRC Reference: ES/S012567/1). C.M. is supported by an ERC Consolidator Award (648837 REACH). H.L.F. is supported by a British Academy Mid-Career Fellowship (MD\170005). U.R. is supported by a Heisenberg professorship from the German Research Foundation (grant no. 389624707).

## Supplementary Material

sbaa005_suppl_Supplementary_Tables

sbaa005_suppl_Supplementary_Material
