# Supplementary material for: Threatening Life Events and Difficulties and Psychotic Disorder
Source: Schizophr Bull. 2020 Feb 12;46(4):814–22. doi: 10.1093/schbul/sbaa005 (PMC7342097; doi:10.1093/schbul/sbaa005)
Supplement: sbaa005_suppl_Supplementary_Tables [file sbaa005_suppl_supplementary_tables.docx]

**Supplementary Tables**

**Supplementary Table 1.** Comparison of basic characteristics of CAPsy sample with a concurrent incidence study sample.

|  | | **CAPsy (n, 374)** | | **CRIS (n, 558)*** | |
| --- | --- | --- | --- | --- | --- |
| **Gender** | |  |  |  |  |
|  | Men | 229 | 61.2% | 292 | 52.3% |
|  | Women | 145 | 38.8% | 266 | 47.7% |
| **Age** | |  |  |  |  |
|  | Mean (SD) | 28.9 | 8.9 | 33.3 | 10.7 |
| **Ethnicity** | |  |  |  |  |
|  | White British | 92 | 24.9% | 133 | 23.8% |
|  | White Other | 46 | 12.4% | 147 | 26.3% |
|  | Black African | 94 | 25.4% | 75 | 13.4% |
|  | Black Caribbean | 60 | 16.2% | 91 | 16.3% |
|  | Asian (all) | 23 | 6.2% | 44 | 7.9% |
|  | Other | 55 | 14.9% | 68 | 12.1% |
| **Diagnosis** | |  |  |  |  |
|  | Non-affective psychosis | 269 | 76.6% | 250 | 93.6% |
|  | Affective psychosis | 82 | 23.4% | 17 | 6.4% |

CAPsy, Childhood Adversity and Psychosis study. CRIS, Clinical Record Interactive Search system. SD, standard deviation. *CRiS diagnoses available for 1 year only (2010; n=267)

Supplementary Table 2. Comparison of socio-demographic characteristics between LEDS completers and non-completers (cases only).

|  | Completers  (n=253) | Non-completers (n=105) | Non-completers (childhood onset) (n=16) |
| --- | --- | --- | --- |
|  | **Mean (SD)** | **Mean (SD)** | **Mean (SD)** |
| Age in years | 29.0 (8.85) | 29.1 (9.03) | 26.4 (9.24) |
|  | **n (%)** | **n (%)** | **n (%)** |
| Sex |  |  |  |
| Men | 156 (61.7) | 61 (58.1) | 12 (75.0) |
| Women | 97 (38.3) | 44 (41.9) | 4 (25.0) |
| Ethnicity *(4 missing values - 4 non-completers)* | | | |
| White British | 70 (27.7) | 16 (15.8) | 6 (37.5) |
| White Other | 32 (12.7) | 13 (12.9) | 1 (6.3) |
| Black African | 65 (25.7) | 24 (23.8) | 5 (31.3) |
| Black Caribbean | 45 (17.8) | 13 (12.9) | 2 (12.5) |
| Asian (all) | 13 (5.1) | 10 (9.9) | 0 (0.0) |
| Other | 28 (11.1) | 25 (24.8) | 2 (12.5) |
| Highest level of education *(29 missing values – 4 completers, 25 non-completers)* | | | |
| Higher | 56 (22.5) | 11 (13.8) | 2 (12.5) |
| Further | 104 (41.8) | 40 (50.0) | 6 (37.5) |
| School | 89 (35.7) | 29 (36.3) | 8 (50.0) |
| Diagnosis *(23 missing values - 3 completers, 20 non-completers)* | | | |
| Non-affective psychosis | 190 (76.0) | 65 (76.5) | 14 (87.5) |
| Affective psychosis | 60 (24.0) | 20 (23.5) | 2 (12.5) |

df, degrees of freedom. SD, standard deviation. (Percentages may not add up to 100 due to rounding).

Supplementary Table 3. Prevalence of moderate/marked life events and difficulties in cases with non-affective psychosis, cases with affective psychosis, and controls.

|  | Cases:  non-affective  (n= 190) | Cases:  affective  (n= 60) | Controls  (n=301) |  |  |  |
| --- | --- | --- | --- | --- | --- | --- |
|  | n (%) | n (%) | n (w%) | *x²*† | df † | *p*† |
| Event |  |  |  |  |  |  |
| None | 99 (52.1) | 30 (50.0) | 237 (78.5) | 44.16 | 2 | <0.001 |
| 1 or more | 91 (47.9) | 30 (50.0) | 64 (21.5) |  |  |  |
| Difficulty |  |  |  |  |  |  |
| None | 90 (47.4) | 25 (41.7) | 227 (74.4) | 47.16 | 2 | **<0.001** |
| 1 or more | 100 (52.6) | 35 (58.3) | 74 (25.6) |  |  |  |

w, weighted (for the population proportions of age, gender and ethnicity according to Census values within Lambeth & Southwark). df, degrees of freedom. †, calculated using weights. (Percentages may not add up to 100 due to rounding).

**Supplementary Table 4.** Association between moderate/marked life events and difficulties, by diagnosis.

|  | Weighted unadjusted OR (95% CI) | Weighted adjusted OR‡ (95% CI) |
| --- | --- | --- |
| Life events |  |  |
| Non-affective cases vs. controls | 3.35 (2.18-5.13)*** | 3.42 (2.08-5.63)*** |
| Affective cases vs. controls | 3.64 (2.00-6.63)*** | 3.68 (1.87-7.23)*** |
| Affective vs. non-affective cases | 1.09 (0.61-1.95) | 1.07 (0.57-2.03) |
| Severe difficulties |  |  |
| Non-affective cases vs. controls | 3.23 (2.14-4.88)*** | 4.72 (2.83-7.86)*** |
| Affective pcases vs. controls | 4.07 (2.24-7.37)*** | 4.88 (2.58-9.23)*** |
| Affective vs. non-affective cases | 1.26 (0.70-2.27) | 1.03 (0.56-1.92) |

Weighted for the population proportions of age, gender and ethnicity according to Census values within Lambeth & Southwark. OR, odds ratio. CI, confidence interval. *p<0.05; **p<0.01; ***p<0.001. ‡, adjusted for age, gender, ethnicity, social class.

**Supplementary Table 5.** Association between moderate/marked life events and difficulties (inc. by intrusiveness) and psychotic disorder, adjusted for additional confounders.

|  | Cases (n=178)  *n* (%) | Controls (n=270)  *n* (w%) | Unadjusted OR (95% CI) | Adjusted OR† (95% CI) |
| --- | --- | --- | --- | --- |
| Event |  |  |  |  |
| None | 92 (51.7) | 216 (78.8) | 1 | 1 |
| 1 or more | 86 (48.3) | 54 (21.2) | 3.74 (2.33-6.02)*** | 5.26 (2.75-10.06)*** |
| Difficulty |  |  |  |  |
| None | 84 (47.2) | 205 (74.7) | 1 | 1 |
| 1 or more | 94 (52.8) | 65 (25.3) | 3.94 (2.49-6.25)*** | 7.33 (3.86-13.90)*** |
| Event |  |  |  |  |
| None | 92 (51.7) | 216 (78.8) | 1 | 1 |
| Non-intrusive | 54 (30.3) | 47 (18.7) | 2.80 (1.66-4.70)*** | 4.59 (2.28-9.21)*** |
| Intrusive | 32 (18.0) | 7 (2.5) | 10.29 (4.03-26.30)*** | 8.20 (2.45-27.40)*** |
| Difficulty |  |  |  |  |
| None | 84 (47.2) | 205 (74.7) | 1 | 1 |
| Non-intrusive | 73 (41.0) | 63 (24.5) | 3.21 (1.99-5.17)*** | 6.59 (3.37-12.87)*** |
| Intrusive | 21 (11.8) | 2 (0.8) | 24.46 (5.40-110.81)*** | 19.26 (4.83-76.72)*** |

w, weighted (for the population proportions of age, gender and ethnicity according to Census values within Lambeth & Southwark). OR, odds ratio, calculated using weighted data. CI, confidence interval. *p<0.05; **p<0.01; ***p<0.001. † adjusted for age, gender, ethnicity, social class, current cannabis use, family history of psychosis, and premorbid adjustment (academic and social). (Percentages may not add up to 100 due to rounding).
