# Supplementary material for: Threatening Life Events and Difficulties and Psychotic Disorder
Source: Schizophr Bull. 2020 Feb 12;46(4):814–22. doi: 10.1093/schbul/sbaa005 (PMC7342097; doi:10.1093/schbul/sbaa005)
Supplement: sbaa005_suppl_Supplementary_Material [file sbaa005_suppl_supplementary_material.docx]

Supplementary Material

Appendix 1 Examples of Events and Difficulties

**Examples of threatening intrusive events and difficulties**

001. Event rated ‘marked’ for threat and for intrusiveness

After several failed attempts to claim asylum status, subject was apprehended without any warning, and taken to a detention centre for the first time, where he stayed for over 2 months. He was not allowed to take with him any of his belongings, nor medication for his long-term health problems.

002. Event rated ‘marked’ for threat and for intrusiveness

Mother has a long history of taking drugs and was arrested on suspicion of murder a few weeks earlier. Subject was at home when police came to arrest his mum (who was at work at the time). They searched the home for evidence. Subject had to call his mum to ask her to come home.

003. Difficulty rated ‘low marked’ for threat and for intrusiveness

Since subject got a member of a local gang arrested, after they stole from him and beat him up, subject has since been regularly assaulted by gang members (2-3 times a month). Worst occasion was when perpetrators kicked down door to his property, and beat the subject up, kicked his head, until he passed out. Neighbour had called the police, and subject was woken by the police entering the property. Subject was admitted to hospital for injuries.

**Examples of non-threatening intrusive events and difficulties**

004. Event rated ‘some’ for threat and ‘moderate’ for intrusiveness

Police raided subject’s home to collect evidence against the subject’s brother, who was subsequently arrested for possession of firearms. Whilst the house was being raided, police took away Subject’s personal laptop (which he never retrieved) for evidence.

005. Event rated ‘some’ for threat and ‘moderate’ for intrusiveness

Landlord threatened to evict subject, her husband and their children from the flat, as they were having difficulty paying rent that month. Subject was successful in convincing her landlord to let them stay.

**Examples of threatening non-intrusive events and difficulties**

006. Difficulty rated ‘high moderate’ for threat and ‘none’ for intrusiveness

Subject lives at home with his mum, who excessively uses drugs (crack cocaine). This intensified during the time period relevant to the LEDs interview. She takes drugs in front of him, is often intoxicated, and has started selling her belongings to afford the drugs. Subject and his mum argue frequently about her drug abuse.

007. Event rated ‘marked’ for threat and ‘none’ for intrusiveness

Very close friend had, for a long time, complained of feeling unwell. He went for a check-up, was admitted to hospital, and died unexpectedly the following day from a heart problem. Subject had spent some time with him 7-10 days before he died. Subject shared the expenses of the funeral among friends of the deceased.

**Examples of non-threating, non-intrusive events and difficulties**

008. Event rated ‘some’ for threat and ‘none’ for intrusiveness

Subject moved out of home for the first time to start his undergraduate degree and moved into student accommodation.

009. Difficulty rated ‘mild’ for threat and ‘none’ for intrusiveness

Mum has on-going depression, started several years ago, no attempted suicide in period. Little change in functioning for subject.
